# Psychometrics of three Swedish physical pediatric item banks from the Patient-Reported Outcomes Measurement Information System (PROMIS)®: pain interference, fatigue, and physical activity

**DOI:** 10.1186/s41687-021-00382-2

**Published:** 2021-10-12

**Authors:** Frida Carlberg Rindestig, Marie Wiberg, John Eric Chaplin, Eva Henje, Inga Dennhag

**Affiliations:** 1 Department of Clinical Science, Child- and Adolescent Psychiatry, Umeå University, 90185 Umeå, Sweden; 2 Department of Statistics, USBE, Umeå University, Umeå, Sweden; 3 Department of Pediatrics, Institute of Clinical Sciences, University of Gothenburg, Gothenburg, Sweden

## Abstract

**Background:**

The Patient-Reported Outcomes Measurement Information System (PROMIS®) aims to provide self-reported item banks for several dimensions of physical, mental and social health. Here we investigate the psychometric properties of the Swedish pediatric versions of the Physical Health item banks for pain interference, fatigue and physical activity which can be used in school health care and other clinical pediatric settings. Physical health has been shown to be more important for teenagers’ well-being than ever because of the link to several somatic and mental conditions. The item banks are not yet available in Sweden.

**Methods:**

12- to 19-year-old participants (n = 681) were recruited in public school settings, and at a child- and psychiatric outpatient clinic. Three one-factor models using CFA were performed to evaluate scale dimensionality. We analyzed monotonicity and local independence. The items were calibrated by fitting the graded response model. Differential Item analyses (DIF) for age, gender and language were calculated.

**Results:**

As part of the three one-factor models, we found support that each item bank measures a unidimensional construct. No monotonicity or local dependence were found. We found that 11 items had significant lack of fit in the item response theory (IRT) analyses. The result also showed DIF for age (seven items) and language (nine items). However, the differences on item fits and effect sizes of McFadden were negligible. After considering the analytic results, graphical illustration, item content and clinical relevance we decided to keep all items in the item banks.

**Conclusions:**

We translated and validated the U.S. PROMIS item banks pain interference, fatigue and physical activity into Swedish by applying CFA, IRT and DIF analyses. The results suggest adequacy of the translations in terms of their psychometrics. The questionnaires can be used in school health and other pediatric care. Future studies can be to use Computerized Adaptive Testing (CAT), which provide fewer but reliable items to the test person compared to classical testing.

## Background

Physical inactivity has implication both for somatic medical conditions and for mental health in teenagers. Sedentary lifestyle is linked to the development of several medical conditions, such as heart disease and type 2 diabetes [1, 2], that increases the risk of mental health problems and shortens lifespan by 3–5 years [3]. Physical inactivity is also directly linked to psychiatric symptoms and disorders, such as major depressive disorder, independent of somatic medical conditions [4, 5]. Since, adolescence is a critical developmental phase for the establishment of behavioral habits [6], the level of physical activity during this time period may have long term implications for future levels of physical activity [7–9].

Chronic pain, as defined by persisting or recurring pain over 3 months or more [10], occurs in adolescents with prevalence rates up to 30% [7]. Chronic pain is debilitating, and it impacts function of daily life, here described by the concept of pain interference. Many teenagers with chronic pain, complain about fatigue [9]. Fatigue, in a clinical sense, is defined as an overwhelming, incapacitating, and sustained sense of exhaustion that diminishes one’s ability to perform daily activities [11]. It is a subjective feeling of tiredness which can be either acute or chronic. Prevalence rates vary from 2 to 21% in this age-group [8, 9]. Fatigue can be described conceptually as the experience of fatigue or as the impact of fatigue on physical capacity, cognitive function, and social activities [11, 12]. In this article we use the latter concept of fatigue.

Chronic pain and fatigue often emerge at the onset of puberty and are often linked to a decrease of physical activity, creating a multi-directional causal relationship [13]. We conclude that it is important to monitor physical activity, chronic pain and fatigue in schools [10, 14] and in pediatric clinical settings [5, 15], and to provide validated measures of all three constructs for safer diagnostics and treatments.

The National Institute of Health (NIH) has identified a need for patient-reported outcomes measures that are better validated, more dynamic, and developed with modern test-methodology (www.healthmeasures.net). The pediatric Patient-Reported Outcomes Measurement Information System (PROMIS®) item banks were initially developed through an extensive review of research, expert review of items, qualitative methods with focus groups reviewing items [16] and cognitive interviewing of children [17]. The PROMIS item banks of pain interference [18–20], fatigue [21, 22], and physical activity [23–25] have recently been implemented internationally [23, 26, 27], but are not yet available in Sweden.

Several pediatric scales have been developed by using classical test theory to measure pain (i.e. The Faces Pain Scale-Revised [28]), fatigue (i.e. Functional Assessment of Chronic illness Therapy-Fatigue—pedsFACIT-F [29]) and physical activity (i.e. Physical Activity questionnaire for Older Children PAC-C [30]). Modern test-methodology, such as item response theory (IRT), has recently been introduced [21, 23, 29], including the calibration of items and patients onto the same metric, regardless of which latent trait is being measured. Contrary to when classical methods are used, precision measurement may only require a few items to measure a construct because the calibration or weighting of the question is built into the results. In a computer adapted system (CAT) an answer to one question is used to identify the next question to be asked that will reduce the error rate of the predicted total score. By using CAT respondents do not need to report on the same items as each other in order to produce comparable scores. Different questions within the same item bank can be used to arrive at a total score for that domain. Thus IRT techniques minimize the number of items presented to each respondent and further prevent test-tiredness by the possibility of answering different questions at each test occasion.

This study is part of a Swedish PROMIS cooperative research group [31] aiming to translate and standardize PROMIS measures across global initiatives and settings. We work to create a shared unified terminology and metric to report common symptoms and functional life domains. PROMIS item banks offer great potential for improving Swedish and global assessment in clinical trials and evaluation of treatment and health care in clinical settings.

In this study, we validated the Swedish translations of three PROMIS Pediatric item banks. The PROMIS pediatric scale of pain interference has been used in studies among child and adolescent populations such as juvenile fibromyalgia and sickle cell disease [17–20, 32] and shown good psychometric properties. The PROMIS pediatric *Fatigue* has previously been applied in several studies of child- and adolescent populations [20–22]. One article using IRT, Lai et al. [21], showed that the scale *Fatigue* demonstrated satisfactory psychometric properties after removing two items. The PROMIS pediatric *Physical activity* [23–25], has also previously shown to be a precise and valid measurement of children’s lived experiences of physical activity [23].

The Swedish versions of the item banks need to be validated to ensure that quality and consistency are maintained from the PROMIS original English versions. The aim of this study was to validate three item banks in a Swedish population: The PROMIS pediatric item banks of Pain Interference v.2.0, Pediatric Fatigue v.2.0 and Pediatric Physical Activity v.1.0. These item banks were recently translated to Swedish [21].

## Methods

### Study setting

The study was conducted in the northern part of Sweden and was approved by the Regional Swedish Ethical Review Board in Umeå (number 2018/59-31). The authors have been working with PROMIS Health Organization since 2016. Authorization to translate the item banks was granted in the fall of 2016.

### Procedure

Adolescents (*n* = 681) were recruited between September 2018 and May 2019 from four community high schools (*n* = 638) and one child- and adolescent psychiatric (CAP) clinic (*n* = 43). To be eligible for the study, participants had to be fluent in spoken and written Swedish. Oral and written informed consent was gathered from participants and their parents (for children under 15 years).

All participants completed the survey on-line during approximately 30–45 min, and they received a gift card for their participation.

### Participants

High-school students (n = 897) and CAP patients (n = 160) were asked to participate and 71% of the high-school students (n = 638) and 27% (n = 43) of the CAP clinic patients agreed to participate, which rendered a total sample of 419 girls and 262 boys between 12 and 19 years of age (M = 15.75, SD = 1.77). Most participants were of Swedish origin (91%). The socioeconomic status of the households was distributed as follows: 17% manual workers, 28% clerical or office workers, 32% higher civil servants, and executives, 7% self-employed of different kinds, 1% students, and 15% unknown. A subset of the adolescents (n = 238 girls and n = 110 boys, mean age 15.39, SD = 1.68) was invited for retesting approximately 3 weeks after the first assessment.

### US sample for DIF analyses

For comparative analyses of language, a US sample [33] was used in the DIF analyses. From which only the variables that we analyzed in the present article was extracted. US data was only available for the pain and fatigue PROMIS item banks. The sample consisted of N = 356 adolescent (173 girls) between 12 and 17 years of age, (M = 14.70, SD = 1.72). All participants suffered from different medical conditions (19% cancer, 40% kidney problems, 15% rheumatic conditions, and 26% sickle cell anemia). The sample has been described in further detail elsewhere [33].

### Translation and adaption of the item banks

**Fig. 1:**
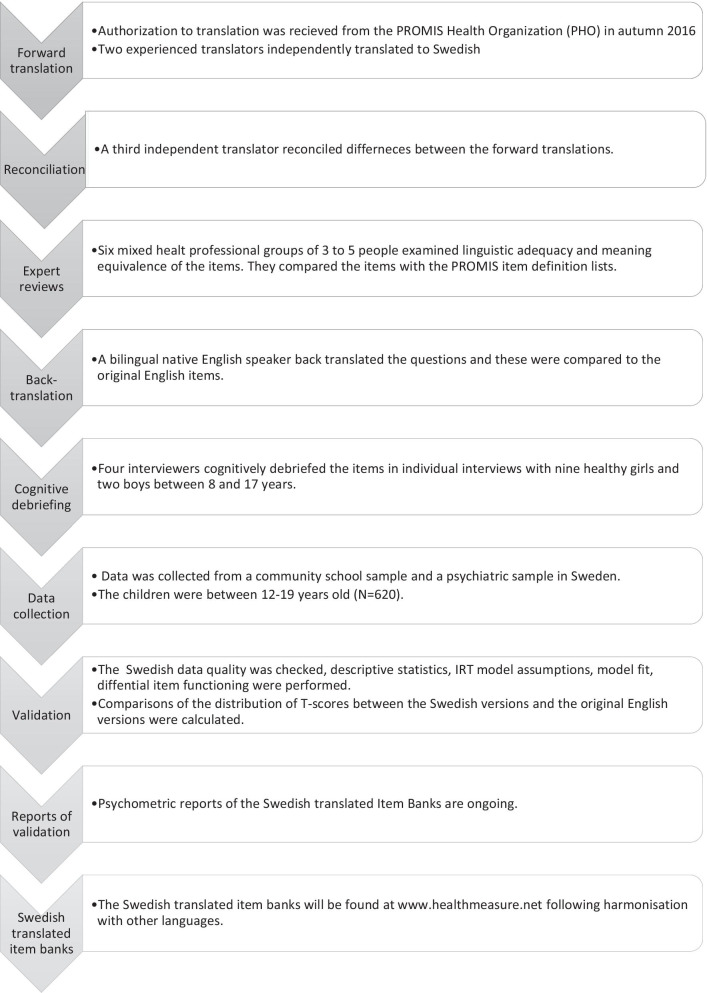
The translation process from the PROMIS item banks to the Swedish translated item banks

Functional Assessment of Chronic Illness Therapy (FACIT) Multilingual Translation Methodology [34, 35], with some modifications, was used for translation. Forward translation, reconciliation, expert reviews, back-translation, cognitive debriefing, and pilot testing were performed. For more details, see Blomqvist et al. [29, 31]. See Fig. 1, for an overview of the Swedish translation and adaption processes. The current translated item banks are found in the step “Reports of validation” in Fig. 1.

### Self-report instruments

#### PROMIS

Patient Reported Outcome Measurements Information System consists of item banks measuring generic health [12]. In the present study, the item banks for pain interference, fatigue, and physical activity were used.

#### PROMIS Pediatric Pain Interference v.2.0. [36]

The pain interference questionnaire measures the perceived extent to which pain has disrupted daily living over the last 7 days. It consists of 20 questions on a 5-point summated-rated scale ranging from 1 (never) to 5 (almost always).

#### PROMIS Pediatric Fatigue v.2.0. [12]

The fatigue questionnaire measures how tired the child has felt during the last 7 days. The 25 questions are rated on a 5-point scale ranging from 1 (never) to 5 (almost always).

#### PROMIS Pediatric Physical Activity v.1.0. [23, 25]

The physical activity questionnaire measured how much physical activity the child has had during the last 7 days. The 10 questions are rated on a 5-point scale of 1 (no days), 2 (1 day), 3 (2–3 days), 4 (4–5 days), and 5 (6–7 days) except for one item (On a usual day, how physically active were you?) that was answered with 1 (Not at all), 2 (A little bit), 3 (Somewhat), 4 (Quite a bit), or 5 (Very much).

### Statistical and psychometric methods

The analyses were performed in IBM SPSS, Version 26.0 and in R [37]. Psychometric calculations followed the method described in Reeve et al., [38]. First, descriptive statistics was calculated. Thereafter, corrected item-total correlations (*r*_it_^c^) was estimated. A correlation less than 0.3 indicates that the corresponding item does not correlate well with the overall scale and should be removed [39]. The reliability of the scales were calculated using Cronbach’s α (good internal consistency is proposed to be between 0.70 and 0.90 [40]. Further IRT Test Information Function (TIF), Item Information Curves (IIC) and Standard Errors (SE) were calculated. TIF is inversely related to SE. A SE of 0.32 corresponds to a reliability of 0.90 according to the formula: r = 1–SE^2^, e.g. 1–0.3^2^ = 1–0.09 = 0.91 [41], the smaller SE the better reliability.

We performed a test–retest analysis, with 3 weeks between the tests, and correlations were measured through intraclass correlation coefficients (ICCs), with a two-way fixed effects model [42]. Values below 0.40 were considered poor, from 0.40 to 0.75 were fair to good, and values greater than 0.75 were excellent according to the criteria of Fleiss [43].

#### Unidimensionality

Before using IRT, we checked for unidimensionality (all items must load on a single factor) in the item banks with three single factor Comparative Factor Analyses (CFA) of the inter-item polychoric correlation matrices (as recommended by Reeve [38]. Due to the non-normal distribution found in the data and the use of ordinal data, we used the diagonally weighted least squares estimator with robust standard error [44] in the R package Lavaan for structural equation modeling version 0.6-3 [45]. Goodness of fit indices used in the study were Comparative Fit Index (CFI), Tucker Lewis Index (TLI), Root Means Square Error of Approximation (RMSEA) and Standardized Root Mean Residual (SRMR). We followed the recommendations form Hu and Bentler [46] and PROMIS analysis plan [38] for unidimensionality CFI > 0.95, TLI > 0.95, RMSEA < 0.06 and SRMR < 0.08.

#### Monotonicity and local independence

We assessed monotonicity and local independence using a non-parametric IRT model with Mokken scale analyses using R-package Mokken (version 3.0.3) [47]. Coefficients of homogeneity (H) were examined and monotonicity was indicated with item values at 0.3 or above and for total scale values at least 0.50 [48]. Local independence was checked by conditional association and reported with true/false values, if all values are true the items show local independence.

#### Graded response models

In addition, the items were fitted with the graded response model [49] with the R package ltm [50]. The discrimination (slope) and difficulty (thresholds) were calculated for each item. The four threshold parameters (beta coefficients for five alternative answers) were used to indicate the level of pain interference, fatigue, and physical activity at which a response in a particular category becomes likely. The goodness of fit of the IRT model (item-fit) was examined using S-χ^2^ statistic for polytomous response data [51]. A non-significant value indicated adequate fit of the model to the data (p > 0.001 [52]).

#### Differential item function (DIF)

DIF for gender, age (median split), language (Swedish translated vs US original pediatric PROMIS item banks of pain and fatigue) [33], were calculated for each item on each scale using the IRT Likelihood Ratio DIF approach [53], using LR χ^2^ item fit statistics, as implemented in the software R package mirt [54]. The Benjamini–Hochberg procedure [55] was used to control for multiplicity of comparisons in DIF (see Table 2). McFadden’s R^2^ was used to evaluate when DIF was detected (> 2%) [40]. McFadden’s R^2^ could be interpreted as < 0.035 = negligible DIF, 0.035–0.07 = moderate DIF, and > 0.07 = large DIF [56].The level of the effect size was evaluated tabular and graphically using methods outlined by Steinberg and Thissen [57] for items with significant DIF.

We transformed the theta scores into *T*-scores as recommended by PROMIS using the formula ((θ*10) + 50. The average *T*-score of the study population is 50 (SD = 10).

## Results

### Descriptive statistics and confirmatory factor analysis

The data showed good range and response distribution within the items. Descriptive statistics are shown in Table 1. Missing data analysis was performed and showed 0.3% missing data in all three item banks respectively. Missing data were replaced with imputed values using linear regression. Data was assumed to be missing at random.

**Table 1:** Descriptive statistics for the Swedish translated PROMIS Pediatric item banks Pain Interference v.2.0., Pediatric Fatigue v.2.0., and Pediatric Physical Activity v.1.0

Items	Total sample *N* = 681				Response Category Frequencies				
	Mean	SD	*r*_it_^c^	*α-i*	1	2	3	4	*5*
*Pain interference total scale*	29.24	14.28							
1. I felt angry when I had pain …	1.58	0.98	0.70	0.97	458	102	85	20	16
2. I had trouble doing schoolwork when I had pain …	1.68	1.08	0.82	0.97	443	94	90	30	24
3. I had trouble sleeping when I had pain…	1.65	1.06	0.80	0.97	448	100	81	30	22
4. It was hard for me to pay attention when I had pain …	1.69	1.08	0.84	0.97	435	97	94	34	21
5. It was hard for me to run when I had pain …	1.79	1.18	0.76	0.97	416	99	93	37	36
6. It was hard for me to walk one block when I had pain …	1.39	0.82	0.80	0.97	523	80	56	13	9
7. It was hard to have fun when I had pain…	1.57	1.01	0.82	0.97	477	83	75	29	17
8. It was hard to stay standing when I had pain …	1.46	0.93	0.84	0.97	513	74	57	24	13
9. I hurt a lot …	1.59	0.97	0.82	0.97	452	111	80	23	15
10. I hurt all over my body…	1.42	0.84	0.74	0.97	509	93	55	15	9
11. I missed school when I had pain …	1.31	0.69	0.77	0.97	542	82	46	7	4
12. It was hard for me to remember things when I had pain …	1.32	0.76	0.79	0.97	547	78	39	7	10
13. It was hard to get along with other people when I had pain …	1.32	0.73	0.80	0.97	542	78	45	11	5
14. It was hard for me to be away from home because I had pain …	1.33	0.75	0.83	0.97	546	70	48	11	6
15. It was hard to have fun with friends because I was in pain…	1.37	0.80	0.84	0.97	531	77	50	16	7
16. I needed help walking when I was in pain …	1.21	0.61	0.71	0.97	592	51	28	6	4
17. I walked carefully when I was in pain…	1.49	0.95	0.75	0.97	496	88	60	21	16
18. I had so much pain I had to stop what I was doing…	1.39	0.82	0.81	0.97	528	71	58	19	5
19. My pain was so bad that I needed to take medicine to treat it…	1.41	0.89	0.67	0.97	529	69	50	20	13
20. It was hard to do things with my family because I had pain …	1.27	0.71	0.78	0.97	571	59	34	10	7
*Fatigue total scale*	52.63	22.77							
1. Being tired made it hard for me to keep up with my schoolwork…	2.36	1.27	0.78	0.97	235	152	159	84	51
2. Being tired made it hard for me to play or go out with my friends as much as I'd like…	1.86	1.11	0.73	0.97	364	134	117	44	22
3. I felt weak…	2.04	1.16	0.74	0.97	306	154	135	60	26
4. I got tired easily…	2.74	1.32	0.74	0.97	163	139	167	135	77
5. I had trouble finishing things because I was too tired…	2.39	1.27	0.79	0.97	233	139	169	93	47
6. I had trouble starting things because I was too tired…	2.52	1.32	0.79	0.97	219	125	163	115	59
7. I was so tired it was hard for me to pay attention…	2.31	1.22	0.81	0.97	246	133	177	93	32
8. I was too tired to do sports or exercise…	2.11	1.28	0.72	0.97	304	154	119	49	55
9. I was too tired to do things outside…	1.84	1.08	0.79	0.97	365	142	104	58	12
10. I was too tired to enjoy the things I like to do…	1.89	1.10	0.80	0.97	353	132	133	45	18
11. I felt tired even when I had not done anything…	2.29	1.30	0.81	0.97	274	118	150	93	46
12. It was hard for me to get out of bed in the morning because I was too tired …	2.81	1.44	0.68	0.97	184	114	143	25	115
13. I felt too tired to spend time with my friends…	1.85	1.06	0.77	0.97	347	160	118	39	17
14. I felt more tired than usual when I woke up in the morning…	2.31	1.30	0.74	0.97	260	135	159	71	56
15. I felt tired…	3.02	1.36	0.73	0.97	138	95	176	156	116
16. I needed to sleep during the day…	2.15	1.31	0.64	0.97	315	121	132	55	58
17. I was too tired to watch television…	1.63	0.97	0.68	0.97	433	118	93	26	11
18. I was too tired to eat…	1.50	0.91	0.66	0.97	488	86	77	20	10
19. I was too tired to take a bath or shower…	1.70	1.05	0.71	0.97	424	111	90	41	15
20. I was so tired it was hard for me to focus on my work…	2.31	1.25	0.83	0.97	252	134	174	77	44
21. Being tired kept me from having fun…	1.72	1.02	0.80	0.97	401	134	97	36	13
22. I was too tired to go up and down a lot of stairs…	1.59	1.03	0.68	0.97	464	106	60	28	23
23. I was too tired to go out with my family…	1.58	0.97	0.71	0.97	451	124	65	25	16
24. I was too tired to read…	2.15	1.28	0.74	0.97	310	121	134	72	44
25. Being tired made it hard for me to remember things…	1.97	1.17	0.80	0.97	339	133	123	61	25
*Physical activity total scale*	29.73	9.07							
1. How many days did you exercise so much that you breathed hard?	2.93	1.15	0.84	0.92	102	107	267	145	60
2. How many days did you play sports for 10 min or more?	3.32	1.18	0.77	0.93	67	75	237	179	123
3. How many days were you so physically active that you sweated?	3.10	1.12	0.84	0.93	74	102	259	173	73
4. How many days did you exercise or play so hard that your body got tired?	2.87	1.13	0.85	0.92	105	112	278	135	51
5. How many days did you exercise or play so hard that your muscles burned?	2.49	1.09	0.76	0.93	153	184	224	96	24
6. How many days did you exercise or play so hard that you felt tired?	2.75	1.14	0.78	0.93	123	135	258	121	44
7. On a usual day. how physically active were you?	3.31	1.03	0.63	0.93	23	145	188	249	76
8. How many days did you exercise really hard for 10 min or more?	2.73	1.18	0.81	0.93	133	138	238	122	50
9. How many days were you physically active for 10 min or more?	3.72	1.16	0.52	0.94	38	56	187	178	222
10. How many days did you run for 10 min or more?	2.50	1.21	0.66	0.93	189	141	209	103	39

*M* mean, *SD* standard deviation, *r*_it_^c^ corrected item-total correlation, *α-i* ordinal alpha if the item is removed

Corrected item-total correlations (r_it_^c^) were greater than 0.3 in the total sample (ranging from 0.52 to 0.85) and in the male and female subsamples (0.62 to 0.88 vs. 0.46 to 0.86, respectively). The corresponding items correlated well with the overall scales.

The internal consistency in terms of Cronbach alpha for the three item banks were very high: pain interference (α = 0.97, 95% CI [0.97, 0.97]), fatigue (α = 0.97, 95% CI [0.97, 0.97]) and physical activity (α = 0.94, 95% CI [0.93, 0.94]).

Test consistency over time was calculated using a subsample of n = 348 adolescents (55% of the original sample of N = 638 answered the questionnaire again 3 weeks later). The test–retest ICCs were 0.84 for the total score of the pain interference (95% CI 0.80, 0.87; F = 6.07; p ≤ 0.001), 0.89 for the fatigue (95% CI 0.86, 0.91; F = 9.04; p ≤ 0.001), and 0.86 for the physical activity item bank (95% CI 0.82, 0.88; F = 6.94; p ≤ 0.001). Based on the criteria of Fleiss [43], the ICCs were considered very good.

#### Unidimensionality

Unidimensionality within the scales was concluded from the three performed single CFAs. The results were as follows: χ^2^ (1375) = 2768.09, CFI = 0.98, TLI = 0.98, RMSEA = 0.08, 90% CI [0.07, 0.09], SRMR = 0.05 for pain interference; χ^2^ (275) = 2100.35, CFI = 0.96, TLI = 0.96, RMSEA = 0.10, 90% CI [0.10, 0.10], SRMR = 0.06 for fatigue; and χ^2^ (35) = 626.45, CFI = 0.98, TLI = 0.97, RMSEA = 0.16, 90% CI [0.15, 0.17]), SRMR = 0.04 for physical activity. Goodness of fit indices showed a good fit of the models to the data, except for RMSEA that showed a moderate fit, and a relatively low fit (0.16) for physical activity. The subscales showed standardized factor loadings greater than 0.40 for all items (for pain interference ranging from 0.81 to 0.94; for fatigue ranging from 0.71 to 0.90, and for physical activity ranging from 0.59 to 0.91) (factor loadings are available on request). Moreover, the items were conditionally independent in the model showing no pairs of items with significant residual correlations.

#### Monotonicity and local independence

The basic IRT assumptions were evaluated and showed monotonicity (H for pain interference items ranged 0.59 to 0.70 [total scale H = 0.68], fatigue items ranged 0.53–0.69 [total scale H = 0.63] and physical activity items ranged 0.48–0.72 [total scale H = 0.65]), and local independence was found among the items.

### Graded response models

**Fig. 2:**
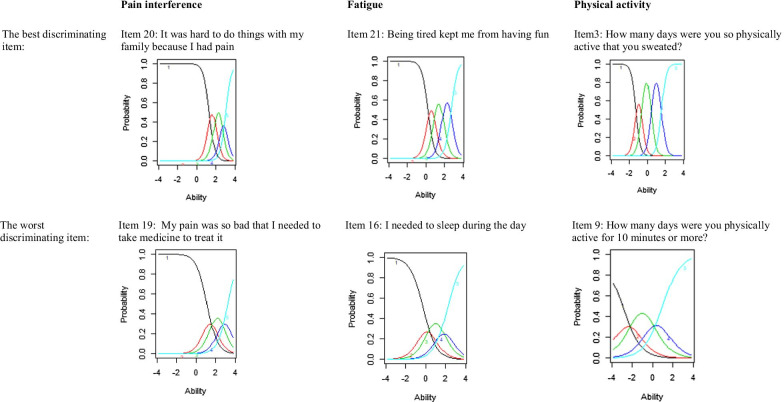
The best and worst discriminating items in the Swedish translated item

The item parameter estimates and the χ^2^ mean square item fit statistics are shown in Table 2. In this table the items are sorted in order of decreasing discrimination (a), so the generally best indicators of pain interference, fatigue, and physical activity are near the top of the tables. The best and the worst discriminating items are shown in category characteristic curves, see Fig. 2.

**Table 2:** Item parameters, item fit index, differential item function and effect size for the Swedish translated item banks: PROMIS Pediatric Pain Interference v.2.0., PROMIS Pediatric Fatigue v.2.0., and PROMIS Pediatric Physical Activity v.1.0

Swedish translated Items	Item parameters					SS X^2^ itemfit index	Differential item functioning											
							Gender SS X^2^ fit index			Age SS X^2^ fit index			Language SS X^2^ fit index			McFadden R^2^ uniform^1^ effect size		
Pain interference	a	b1	b2	b3	b4	p-value	Chisq	df	p-value	Chisq	df	p-value	Chisq	df	p-value	Age	Gender	Language
It was hard to do things with my family because I had pain	3.38	1.32	1.93	2.58	3.03	1.000	7.87	5	.163	1.30	5	.934	17.36	5	.004	.00	.00	.00
It was hard for me to be away from home because I had pain	3.30	1.12	1.76	2.61	3.28	1.000	8.73	5	.121	3.35	5	.647	28.11	5	.000	.00	.00	.00
It was hard to have fun with friends because I was in pain	3.09	1.00	1.67	2.44	3.09	1.000	2.97	5	.704	2.89	5	.716	8.27	5	.142	.00	.00	.00
It was hard to have fun when I had pain	2.96	0.70	1.29	1.98	2.60	0.998	4.46	5	.486	7.69	5	.174	4.85	5	.434	.00	.00	.00
I had so much pain I had to stop what I was doing	2.96	1.03	1.60	2.45	3.22	1.000	2.86	5	.721	4.57	5	.471	4.30	5	.507	.00	.00	.00
It was hard to stay standing when I had pain	2.92	0.91	1.52	2.24	2.88	1.000	3.74	5	.587	1.71	5	.888	4.29	5	.508	.00	.00	.00
It was hard for me to pay attention when I had pain	2.89	0.44	1.03	2.03	2.70	0.998	6.66	5	.247	8.25	5	.143	48.49	5	.000*	.00	.00	.02
It was hard to get along with other people when I had pain	2.88	1.11	1.84	2.71	3.53	1.000	7.62	5	.178	8.64	5	.124	23.11	5	.000	.00	.00	.00
It was hard for me to remember things when I had pain	2.76	1.14	1.89	2.71	3.01	0.970	6.51	5	.260	1.40	5	.924	32.23	5	.000	.00	.00	.01
I had trouble doing schoolwork when I had pain	2.72	0.48	1.09	1.93	2.48	0.904	1.21	5	.944	4.47	5	.484	64.97	5	.000*	.00	.00	.02
It was hard for me to walk one block when I had pain	2.72	0.98	1.63	2.49	2.99	0.987	4.99	5	.416	4.84	5	.436	18.73	5	.002	.00	.00	.00
I missed school when I had pain	2.66	1.13	1.90	3.00	3.66	0.479	9.15	5	.103	2.12	5	.832	44.88	5	.000*	.00	.00	.01
I hurt a lot	2.50	0.59	1.33	2.23	2.82	0.823	5.84	5	.322	2.25	5	.813	31.81	5	.000	.00	.00	.01
I had trouble sleeping when I had pain	2.48	0.56	1.26	2.10	2.71	0.824	3.65	5	.601	5.19	5	.392	15.52	5	.008	.00	.00	.00
I needed help walking when I was in pain	2.46	1.55	2.18	3.01	3.72	1.000	4.68	5	.456	14.82	5	.011*	12.45	5	.029	.00	.00	.01
It was hard for me to run when I had pain	2.28	0.40	1.04	1.79	2.31	0.000	4.39	5	.495	5.43	5	.365	3.57	5	.613	.00	.00	.00
I walked carefully when I was in pain	2.16	0.89	1.56	2.38	2.95	0.000	2.90	5	.715	11.15	5	.048	31.71	5	.000	.00	.00	.01
I felt angry when I had pain	2.02	0.64	1.40	2.37	2.94	0.000	9.81	5	.081	8.06	5	.153	16.32	5	.006	.00	.00	.00
I hurt all over my body	1.97	0.97	1.81	2.80	3.47	0.000	9.97	5	.076	2.84	5	.724	43.08	5	.000*	.00	.00	.02
My pain was so bad that I needed to take medicine to treat it	1.88	1.14	1.79	2.58	3.23	0.000	0.94	5	.967	10.92	5	.053	29.86	5	.000	.00	.01	.01
Fatigue																		
Being tired kept me from having fun	2.95	0.25	0.98	1.84	2.72	1.000	8.89	5	.114	5.53	5	.355	117.08	5	.000*	.00	.00	.04
I was so tired it was hard for me to focus on my work	2.83	− 0.51	0.17	1.17	1.94	1.000	2.70	5	.746	2.45	5	.784	10.31	5	.037	.00	.00	.00
I was too tired to enjoy the things I like to do	2.74	0.01	0.69	1.67	2.55	1.000	7.05	5	.217	1.60	5	.901	2.64	5	.076	.00	.00	.00
I was so tired it was hard for me to pay attention	2.56	− 0.56	0.16	1.16	2.16	1.000	5.79	5	.328	18.58	5	.002*	9.88	5	.0.79	.01	.00	.00
I felt tired even when I had not done anything	2.55	− 0.39	0.23	1.06	1.94	1.000	6.42	5	.267	5.13	5	.401	19.45	5	.002	.00	.00	.00
Being tired made it hard for me to remember things	2.52	− 0.08	0.64	1.48	2.38	1.000	6.75	5	.240	5.42	5	.367	11.60	5	.041	.00	.00	.00
I felt too tired to spend time with my friends	2.46	− 0.02	0.84	1.82	2.68	1.000	9.41	5	.094	5.06	5	.408	3.61	5	.607	.00	.00	.00
I was too tired to do things outside	2.46	0.07	0.84	1.64	2.89	1.000	6.52	5	.259	7.93	5	.160	63.27	5	.000*	.00	.00	.02
I had trouble finishing things because I was too tired	2.37	− 0.66	0.11	1.06	1.95	1.000	7.54	5	.183	9.03	5	.108	47.25	5	.000	.00	.00	.02
I had trouble starting things because I was too tired	2.30	− 0.73	− 0.03	0.88	1.83	0.997	7.16	5	.209	18.32	5	.003*	119.71	5	.000*	.01	.00	.04
I was too tired to go out with my family	2.25	0.53	1.32	2.08	2.78	1.000	9.34	5	.096	4.04	5	.544	24.39	5	.000	.00	.00	.01
Being tired made it hard for me to play or go out with my friends as much as I'd like	2.11	0.06	0.81	1.76	2.60	0.200	1.12	5	.952	3.23	5	.664	26.38	5	.000	.00	.00	.01
I was too tired to read	2.09	− 0.22	0.44	1.31	2.10	0.005	10.09	5	.073	6.10	5	.296	6.56	5	.255	.00	.00	.00
Being tired made it hard for me to keep up with my schoolwork	2.07	− 0.67	0.17	1.17	2.02	0.041	1.52	5	.911	3.22	5	.667	29.90	5	.000	.00	.00	.01
I was too tired to go up and down a lot of stairs	2.06	0.62	1.33	1.99	2.59	0.996	2.65	5	.753	12.64	5	.027	108.23	5	.000*	.01	.00	.05
I was too tired to eat	2.00	0.76	1.37	2.44	3.26	0.882	2.29	5	.808	4.99	5	.418	14.17	5	.013	.00	.00	.00
I was too tired to take a bath or shower	1.99	0.39	1.07	1.92	2.96	0.986	2.63	5	.756	12.80	5	.025*	8.06	5	.153	.01	.00	.00
I got tired easily	1.99	− 1.12	− 0.25	0.68	1.70	0.087	6.41	5	.268	6.56	5	.255	30.70	5	.000	.00	.00	.01
I felt weak	1.97	− 0.27	0.58	1.59	2.54	0.008	2.58	5	.765	5.02	5	.413	15.21	5	.010	.00	.00	.00
I felt more tired than usual when I woke up in the morning	1.97	− 0.51	0.25	1.25	1.97	0.402	6.54	5	.257	7.42	5	.191	3.94	5	.558	.00	.00	.00
I felt tired	1.90	− 1.32	− 0.67	0.30	1.34	0.004	3.71	5	.592	3.42	5	.635	52.90	5	.000	.00	.00	.01
I was too tired to watch television	1.88	0.45	1.21	2.33	3.25	0.935	3.20	5	.669	3.62	5	.605	8.47	5	.132	.00	.00	.00
I was too tired to do sports or exercise	1.85	− 0.28	0.58	1.47	2.06	0.000	4.72	5	.451	3.95	5	.557	10.15	5	.071	.00	.00	.00
It was hard for me to get out of bed in the morning because I was too tired	1.65	− 1.00	− 0.23	0.59	1.45	0.000	4.64	5	.461	3.89	5	.565	14.57	5	.012	.00	.00	.00
I needed to sleep during the day	1.49	− 0.22	0.52	1.51	2.18	0.000	7.85	5	.165	18.65	5	.002*	76.34	5	.000*	.01	.00	.01
Physical Activity																		
How many days were you so physically active that you sweated?	3.88	− 1.30	− 0.64	0.47	1.58	1.000	1.15	5	.949	1.28	5	.940				.00	.00	
How many days did you exercise so much that you breathed hard?	3.87	− 1.08	− 0.50	0.58	1.66	1.000	7.12	5	.212	5.84	5	.323				.00	.00	
How many days did you exercise or play so hard that your body got tired?	3.67	− 1.09	− 0.47	0.72	1.82	1.000	2.00	5	.849	11.41	5	.044*				.00	.00	
How many days did you exercise or play so hard that you felt tired?	3.05	− 1.01	− 0.34	0.87	1.95	1.000	4.15	5	.529	3.32	5	.650				.01	.00	
How many days did you exercise really hard for 10 min or more?	3.01	− 0.92	− 0.25	0.82	1.92	1.000	2.45	5	.784	2.38	5	.795				.00	.00	
How many days did you exercise or play so hard that your muscles burned?	2.75	− 0.85	− 0.01	1.17	2.45	0.988	4.35	5	.500	1.46	5	.917				.01	.00	
How many days did you play sports for 10 min or more?	2.67	− 1.54	− 0.88	0.23	1.19	1.000	2.88	5	.219	3.04	5	.690				.00	.00	
How many days did you run for 10 min or more?	1.92	− 0.78	− 0.07	1.12	2.26	0.000	1.03	5	.960	7.85	5	.165				.01	.00	
On a usual day. how physically active were you?	1.60	− 2.62	− 0.96	0.12	1.93	0.000	4.67	5	.457	5.21	5	.391				.00	.00	
How many days were you physically active for 10 min or more?	1.11	− 2.96	− 1.84	− 0.18	1.00	0.000	11.32	5	.045	15.28	5	.009*				.01	.00	

*p is significant after Benjamini–Hochberg correction. The scale for the item parameters is standardized, mean 0 variance 1, as is conventional for reporting IRT parameters. The order is following alpha from large to small in every subscale. ^1^Uniform McFadden effect size is reported and non uniform is available on request

For the pain interference items, five of the items exhibited significant lack of fit as indicated by the SS χ^2^ item fit (p < 0.001, χ^2^ ranged from 503.88 to 754.07, df = 391) (Table 2), after Benjamini–Hochberg correction for multiplicity. For the fatigue items, three of the items showed significant lack of fit (p < 0.05, χ^2^ ranged from 887.04 to 1232.74, df = 636), and for physical activity items, three items showed significant lack of fit (p < 0.05, χ^2^ ranged from 856.52 to 1007.04, df = 662).

**Fig. 3:**
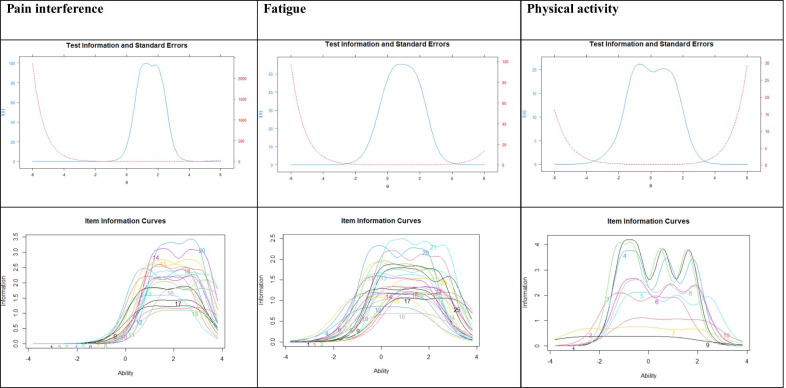
Test information function, standard error, item information curves of the Swedish translated PROMIS item banks

The TIF, IIC, and SE, were satisfactory (see Fig. 3). SE for pain interference items ranged from 0.07 to 0.62 (M = 0.35, SD = 0.68), SE for fatigue items ranged from 0.11 to 0.49 (M = 0.19, SD = 0.70), and SE for physical activity items ranged from 0.16 to 0.52 (M = 0.22, SD = 0.70).

### Differential item function

DIF was used to detect whether gender, age-group and language biased an item. No DIF by gender was found in any of the subscales. For age groups (12–15 years and 16–19 years), there were, after Benjamin Hochberg correction, seven items with significant DIF. One of them had moderate DIF: “I have trouble starting things because I was too tired” (from fatigue item bank). For language (only measured for pain interference and fatigue) there were 9 items with significant DIF after Benjamin Hochberg correction. Most of them had negligible McFadden effect sizes, and only three of the items had moderate DIF (“Being tired kept me from having fun”, “I had trouble starting things because I was too tired”, and “I was too tired to go up and down a lot of stairs” [all three from fatigue item bank]). See Table 2 for the DIF results and the McFadden effect size.

For the items where DIF was found by age and language, we further investigated whether the results were due to the item’s discrimination (slope) or difficulty (thresholds) by using a model where the equal slope assumption was imposed and the difficulty was freely estimated for both of the two groups. There was no significant result for seven items of age, and four items of language. For five items in the item bank fatigue (marked as significant with a star in Table 2 for DIF of language), non-uniformity was found, meaning that the items had different slopes. After considering the analytic results, graphical illustration, item content and clinical relevance we decided to keep all items in the item pools.

The *T*-score calculations were based on the full original English item bank (general and clinical population), obtained from www.assessmentcenter.net/ac_scoringservice. The mean *T*-scores of the study sample were as follows: for pain (M = 46.60, SD = 6.11, range of 42.60–64.20), for fatigue (M = 48.57, SD = 7.77, range of 40.00–63.70) and for physical activity (M = 48.46, SD = 8.44, range of 23.50–72.20). Our T-scores can be provided on request.

## Discussion

One major challenge prior to the use of IRT models is to resolve issues of dimensionality. For all three item banks pain interference, fatigue and physical activity, we found good values on the fit indices CFI, TLI and SRMR. However, for all three item banks, RMSEA values indicated a moderate fit, and for physical activity a relatively low fit (0.16). Values over 0.06 have been reported for many other PROMIS item banks e.g. [41, 58]. Traditional goodness of fit indices has been criticized for not being suitable to establish unidimensionality of health item banks [59] and that RMSEA is sensitive to model complexity and skewed data distributions [59], the latter being the case in our distributions. SRMR has shown to generate more robust results through different populations and estimation methods [60].

Internal consistency or the scale reliability was high in all three item banks (Cronbach’s α ranged from 0.93 to 0.97). The high value of Cronbach’s α is probably partly due to the large number of items included in the scales (and some of the items were quite similar). However, when inspecting the TIF, IIC, and SE curves (IRT) this picture was confirmed but nuanced. At a total mean level, all item banks had satisfied reliability, while at an individual level, the items varied more in reliability. We conclude that the items with low reliability could be set aside in future studies.

Test–retest reliability of the scales and the ICC [43] showed excellent reliability over a period of three weeks (from 0.84 to 0.89 for all subscales). This can be interpreted as very good internal validity and ensures that the scales are both representative and stable over time.

Systematic measurement variability by groups can lead to a number of problems, including errors in hypothesis testing (e.g. it may be assumed that the test covers all genders, all ages or all cultures, but it does not), and misguided research [61]. Ensuring equivalent testing is thus important prior to making comparisons among individuals or groups [61]. We investigated DIF for gender, age-group and language in the three item pools. For all items, no DIF regarding gender was found (not in line with Lai et al. 2013 [21], which found three items due to gender-based DIF), and the subscales measured symptoms equally well for girls and boys. However, some items had DIF regarding age and language, although the effect sizes were mostly negligible (three were moderate for language) and we cannot draw any firm conclusions. DIF by age and language suggests that for these items, depending on age groups (12–15 years and 16–19 years) or language groups (Swedish sample of children speaking Swedish compared with a US sample speaking English), symptoms were not measured very well. For fatigue and age, this was in line with one previous study (Lai et al., 2013 [21], which found that 16 out of 25 fatigue items had DIF for age), while for the other two subscales (pain interference and physical activity) this was a new finding with regard to age. There can be several explanations for this, including that the concept of “fatigue” may not be the same across the age groups. Another potential item bias not measured (because our clinical sample was too small), was DIF regarding psychiatric and physical symptoms; our sample was more normative than the more clinical representation in the US sample.

When comparing the result with our previous review of the translated items (see [31]) we found similarity for only one of the items: “how many days did you run for 10 min or more?”. It was problematic in the translation process because this item is an equivocal item without precise definition in the PROMIS definition list [31, 62]. During cognitive interviews with Swedish children [31, 63], some of them wondered if the item meant that they had done 10 min of continuous running or if the 10 min of running could be accumulated over a day. Even though we translated this item word by word, some children may therefore have interpreted the item differently. DIF by age for this item was not found in the original English version [23]. Several items contained the wording “how many days did you … for 10 min or more” and all of them were in the lower range of all psychometric measurement in our current study as well as in the study by Tucker et al., [23]. Measures of distance and time often need context and a qualitative description to be understandable [64].

A common strategy to deal with DIF items is to set items aside [21]. However, in brief questionnaires this strategy is not recommendable, because it might result in decreased reliability and validity. Apart from that, the shortened scale can lead to a modification of the construct it is intended to measure [65], and removing DIF items in well-established questionnaires decreases comparability between different research studies.

An interesting finding in this study was that the average T-sores of all three item banks was lower than the expected 50.0 (general and clinical US population). This may indicate that Swedish adolescents are, on average, less interfered by pain, less tired, and do less physical activity, compared to US adolescents. However, the samples differ, as our relatively healthy sample overall has less symptoms than the US sample. Further analyses are needed to explore possible alternative explanations.

### Limitations and strengths

The present study had sufficient statistical power and all participants answered all questions, but some limitations should be noted. Participants were not geographically stratified and did not fully match the Swedish general pediatric population, for example, the unbalanced gender ratio limited generalizability. Instead, the participants came from four different schools along with a smaller sample from a child- and adolescent psychiatric clinic. When using IRT statistics, theoretically, a mixed sample is preferable because IRT offers the property of item invariance, in which item parameters are constant even if estimated in different samples [66]. However, our clinical sample was too small to test for DIF and future studies need to investigate if this is also true empirically. For the DIF of language, a sample more similar to ours would have been preferable, as the US sample contained a greater variety of medical diagnoses, which potentially biased the results.

### Implications

The three PROMIS pediatric item banks were translated and adapted to Swedish to meet the need of short, effective and valid tests based on modern test theory such as IRT and DIF for the use in Swedish healthcare [4, 31]. A major advantage in using IRT in health-related outcomes is that it enables adaptive testing, either by multiple short-forms or via computerized adaptive testing [67], which is less of a burden for the patients but not always available in research or clinical settings. Thus, short-forms can be valuable alternatives.

## Conclusions

The PROMIS pediatric item banks of pain, physical activity, and fatigue showed sufficient psychometric properties in a Swedish population. Future studies can be to use Computerized Adaptive Testing (CAT), which provide fewer but reliable items to the test person compared to classical testing (e.g. [41]). This approach prevents test-tiredness.

We hope that the item banks will be implemented both in Swedish school-based health care and in pediatric clinics.

## Data Availability

The data from the current study are available from the corresponding author on reasonable request.

## Abbreviations

CAP: Child- and Adolescent Psychiatry
CFA: Comparative Factor Analysis
CFI: Comparative Fit Index
DIF: Differential item functioning
FACIT: Functional Assessment of Chronic Illness Therapy
ICC: Intraclass correlation coefficient
IRT: Item response theory
NIH: The National Institute of Health
PAC-C: Physical Activity questionnaire for Older Children
PROMIS: Patient-Reported Outcomes Measurement Information System
RMSEA: Root Means Square Error of Approximation
SRMR: Standardized Root Mean Residual
TLI: Tucker Lewis Index
